# Far-infrared irradiation restores mitochondrial dynamics to ameliorate ischemic stroke

**DOI:** 10.4103/NRR.NRR-D-25-00399

**Published:** 2026-01-27

**Authors:** Wanyu Wu, Yuping Wang, Bo Qin, Xiongfei Xu, Yuanqing Qu, Nick Wang, Wuyan Zheng, Xiaoyun Yun, Betty Yuen-Kwan Law, Jianfeng Sun, Wei Zhang, Chang Chen, Vincent Kam-Wai Wong

**Affiliations:** 1 Dr. Neher’s Biophysics Laboratory for Innovative Drug Discovery, State Key Laboratory of Mechanism and Quality of Chinese Medicine, Faculty of Chinese Medicine, Macau University of Science and Technology, Macao Special Administrative Region, China; 2 The Affiliated Traditional Chinese Medicine Hospital of Southwest Medical University, Southwest Medical University, Luzhou, Sichuan Province, China; 3 The Affiliated Hospital of Southwest Medical University, Southwest Medical University, Luzhou, Sichuan Province, China; 4 New Age Technology (Asia) Limited, Hong Kong Special Administrative Region, China; 5 Institute of Chinese Materia Medica, China Academy of Chinese Medical Sciences, Beijing, China

**Keywords:** energy metabolism, far-infrared irradiation, ischemic stroke, middle cerebral artery occlusion (MCAO), mitochondrial dynamics, mitochondrial fusion, neuroprotection, optic atrophy 1 (Opa1), oxidative stress

## Abstract

Far-infrared irradiation exhibits promise in chronic diseases, and its role in ischemic stroke specifically in modulating mitochondrial dynamics remains unknown. This study explored the neuroprotective effects of far-infrared irradiation using a rat middle cerebral artery occlusion model and oxygen-glucose deprivation-injured neuronal cells. In middle cerebral artery occlusion rats, daily 30-minute far-infrared irradiation treatment reduced infarct volume, alleviated cerebral edema, and improved neurological function. Proteomic analysis identified far-infrared irradiation-mediated upregulation of eight proteins, including the mitochondrial fusion regulator optic atrophy 1. In oxygen-glucose deprivation-exposed cells, far-infrared irradiation restored mitochondrial membrane potential, and enhanced fusion via optic atrophy 1 induction. Mechanistically, far-infrared irradiation stabilized mitochondrial dynamics by boosting optic atrophy 1 expression, thereby reducing oxidative stress and maintaining energy production. Optic atrophy 1 knockdown partly abolished the protective effects of far-infrared irradiation therapy. These results establish far-infrared irradiation as a non-pharmacological intervention targeting mitochondrial redox homeostasis in ischemic stroke, offering a novel therapeutic avenue for cerebrovascular disorders.

## Graphical abstract

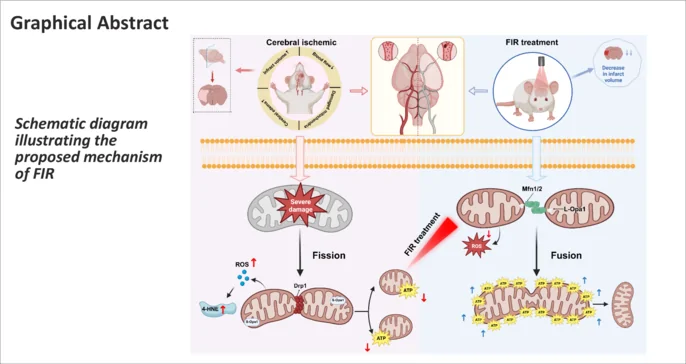

## Introduction

Ischemic stroke (IS), or cerebral infarction, is a widespread affliction worldwide. It is the primary cause of disability and mortality among adults in both developing and industrialized nations (Qin et al., 2022). Ischemic stroke constitutes almost 87% of all stroke occurrences (Li et al., 2026). Clinically, ischemic stroke manifests as sudden facial or limb paralysis, speech disturbances, and visual abnormalities (Liu et al., 2023). As the disease advances, it exhibits a significant disability rate and unfavorable prognosis, resulting in a considerable economic burden on both afflicted people and society (Pu et al., 2023). The predominant therapy for ischemic stroke is intravenous thrombolysis with recombinant tissue plasminogen activator (Zhu et al., 2025). However, patients may experience side effects (Murphy et al., 2023; Bai et al., 2025), with bleeding complications being the main concern (Zhu et al., 2025). These challenges underscore the urgent need for safer, more effective therapeutic strategies.

Ischemic stroke (IS) arises from the cessation of arterial blood flow to the brain and is precipitated by several reasons, including atherosclerosis of major arteries, cardiogenic embolism, and blockage of minor arteries (Hilkens et al., 2024). This interruption leads to oxygen–glucose deprivation (OGD), causing an immediate drop in cerebral blood flow. Consequently, glucose and oxygen utilization are compromised, disrupting ATP synthesis and resulting in energy deficiencies (Li et al., 2024a; Lei et al., 2025). Mitochondria play pivotal roles in maintaining ATP and energy homeostasis. When hypoxia and glucose deficiency disrupt ATP synthesis and energy balance, mitochondrial function and morphology undergo significant alterations. Abnormal processing of optic atrophy 1 (Opa1; e.g., reduction of long-chain isoforms) when ATP synthesis is insufficient leads to loosening of the inner mitochondrial membrane cristae structure and reduced fusion capacity, which in turn triggers mitochondrial fragmentation (Garone et al., 2022; Li et al., 2022b). Mitochondrial malfunction specifically results in the opening of the mitochondrial permeability transition pore (mPTP) (Bonora et al., 2022; Mendoza et al., 2024). Concurrently, reduced ATP levels induce mitochondrial membrane depolarization, marked by alterations in membrane potential (Na^+^ inflow and K^+^ efflux), ultimately resulting in death (Ma et al., 2023). Mitochondrial fusion and fission are two opposing processes that lead to alterations in mitochondrial morphology, with evidence indicating continual variations in mitochondrial structure (Al Ojaimi et al., 2022). The intricate equilibrium between mitochondrial fusion and fission significantly affects mitochondrial homeostasis, cellular stability, and cell viability (Zhang and Petr, 2024; Tábara et al., 2025). While mitochondrial fusion favors cell survival, mitochondrial fission promotes cell death (An et al., 2021). Research has shown that enhancing mitochondrial fusion reduces neuronal apoptosis caused by ischemia-reperfusion damage (Chen et al., 2024; Ge et al., 2024). Moreover, the imbalance in mitochondrial dynamics in the ischemic penumbra is a result of bidirectional regulation of division and fusion (Zhou et al., 2021), and mitochondrial fission and fusion dynamics may be intricately implicated in apoptosis (Kleele et al., 2021).

Sunlight comprises three primary components: ultraviolet, visible, and infrared radiation (Taylor et al., 2022). Among these, infrared radiation can be subdivided into near-infrared, mid-infrared, and far-infrared radiation (FIR). Far-infrared rays pertain to the infrared spectrum with wavelengths spanning from 15 μm to 1 mm (Ozaki, 2022). FIRs possess the unique ability to transfer energy between objects through both thermal and nonthermal effects (Hsu et al., 2019). Indeed, FIRs exhibit strong penetration and radiation capabilities, making them readily absorbable by the human body, where they can be converted into energy (Li et al., 2024b). Moreover, FIRs share a vibrational energy level akin to that of biological macromolecules, allowing them to resonate and exert biological effects (Seo et al., 2021). Recent research has emphasized the effects of FIR on several cell lines, including vascular endothelial cells, neuronal cells, neuroblastoma cells, renal tubular cells, and pancreatic β-cells. These effects include diverse roles, such as inducing angiogenesis, promoting neural repair, enhancing mitochondrial function, and facilitating cell migration (Huang et al., 2012; Chen et al., 2015; Chang et al., 2016; Hsu et al., 2019, 2020). For example, *in vitro* investigations have shown that FIR safeguards from lateral cerebellar paralysis in neurodegenerative illnesses by decreasing polyglutamine accumulation of proteins and enhancing mitochondrial activity (Chang et al., 2016). Furthermore, FIR maintains cellular mitochondrial shape and facilitates mitochondrial fusion by suppressing mitochondrial division. Moreover, FIR radiation has demonstrated the capacity to promote neurite development and regeneration of nerves using neuron-like PC12 cells via AKT1 activation (Wang et al., 2019). In animal research, far-infrared significantly diminishes Aβ deposition and alleviates cognitive impairments in Alzheimer’s disease-like mice via elevating ATP levels (Li et al., 2022a). Additionally, it has been shown that FIR has the ability to enhance the proliferation of rat testicular germ cells in living organisms and guard against testicular ischemia-reperfusion damage by increasing the production of heme oxygenase-1 (Tu et al., 2013), suggesting the potential protective effect of FIR in ischemia-related conditions.

Despite the promising health benefits associated with far-infrared radiation, its specific effects on ischemic stroke remain uncertain. Given that stroke has been closely linked to mitochondrial mass in several prior studies (An et al., 2021; Kleele et al., 2021; Zhou et al., 2021; Ge et al., 2024; Mendoza et al., 2024; Zhang and Petr, 2024; Tábara et al., 2025), we postulated that the impact of FIR on stroke due to ischemia may be associated with mitochondrial function. To evaluate this theory, we examined the effects of FIR therapy on rats subjected to middle cerebral artery occlusion (MCAO). Additionally, we explored how FIR treatment influences mitochondrial function and morphology in MCAO rats. Our study provides evidence of a potential biological effect of FIR treatment on cerebral ischemia rats and sheds light on the protective mechanisms associated with FIR in stroke.

## Methods

### Animals

All Sprague–Dawley (SD) rats were maintained in SPF circumstances at 20–25°C under a 12-hour light–dark cycle, provided a standard rodent diet, and supplied with distilled water. Rats that did not survive before sampling were sampled from the study, and animal body temperatures were maintained between 36.5–37.0°C throughout all anesthesia experiments. Animal data are available from the corresponding author upon reasonable request. Before the start of the experiment, all experimental rats were acclimatized in the same environment.

The Animal Ethics Committee of Macau University of Science and Technology (Macao, China) approved the animal studies on September 5, 2024 (approval No. MUST-20240904001). All animal care and experimental procedures were performed in accordance with the “Institutional Animal Care and Use Committee Guidelines” of the Macau University of Science and Technology. All animal work was conducted according to approved protocols and institutional animal welfare guidelines. Male Sprague–Dawley rats (240–280 g) were procured from Zhuhai Bestest Biotechnology Co. (Zhuhai, China).

**Figure 1:**
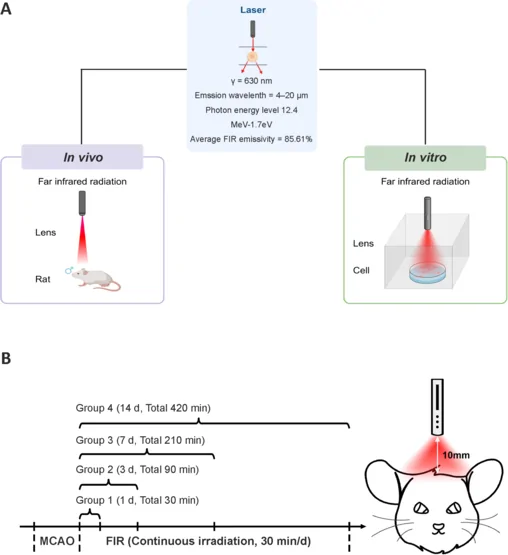
FIR spectrum-emitting device. (A) A range of energy parameters for the FIR radiation device and the setting of FIR irradiation *in vivo* and *in vitro* experiments. Our *in vivo* model utilizes SD rats, while the *in vitro* model employs N-2α cells and Bend3 cells. (B) Schematic illustration of animal experimental timeline (days 1, 3, 7, 14) showing the duration FIR treatment after MCAO in SD rats. FIR: Far-infrared radiation; MCAO: middle cerebral artery occlusion; SD: Sprague–Dawley.

MCAO rats were administered FIR for 30 minutes daily (**Figure 1**). Nimodipine (calcium antagonist) administration was initiated at the time point immediately after awakening of the rats after MCAO and was administered by gavage once a day for 1 day (day 1 group), 3 days (day 3 group), 7 days (day 7 group), and 14 days (day 14 group).

A random selection method was used to randomly assign the rats, and each rat was numbered according to the experimental design group after capture. The researchers did not present selection criteria during the capture process to ensure complete randomization.

Experiment I: Rats were randomly divided into two groups: MCAO and MCAO + FIR, to study survival rate and body weight.

Experiment II: Rats were randomly divided into eight groups: day 1 (MCAO, MCAO + FIR), day 3 (MCAO, MCAO + FIR), day 7 (MCAO, MCAO + FIR), and day 14 (MCAO, MCAO + FIR) to determine the optimal duration for reducing brain infarction and edema. The optimal groups were used for further experiments.

Experiment III: Rats were randomly divided into four groups: Sham, MCAO, Nimodipine (MCAO+Nimodipine 20 mg/kg; Nimodipine, Shyuanye, Shanghai, China), and MCAO+FIR. Neurological effects and cerebral blood flow were assessed using the Longa Neurobehavioral Score and Laser Scatter Hematology. Brain infarction and edema were also measured.

Experiment IV: Rats were randomly divided into three groups: Sham, MCAO, and MCAO + FIR. Proteomic analysis was conducted to identify proteins affected by FIR, with samples from the ischemic cortex collected for validation.

The number of rats included in each experimental group is summarized in **Additional Table 1**.

**Additional Table 1:** Animal allocation in each experiment

Experiment	Groups	Number of rats per group
Experiment I	MCAO, MCAO+FIR	n = 18 per group
Experiment II	Day 1, Day 3, Day 7, Day 14 groups	n ≥ 7 per group
Experiment III	Sham, MCAO, Nimodipine, MCAO+Nimodipine, MCAO+FIR	n ≥ 18 per group
Experiment IV	Sham, MCAO, MCAO+FIR	n ≥ 6 per group

### Far-infrared irradiation spectrum-emitting device

This study utilized the EEFIT LITE® FIR spectrum transmitter (Chen et al., 2021). This apparatus can consistently emit an FIR spectrum with an emission wavelength range of 4–20 μm, a mean photon emissivity of 85.61%, and a photon energy level of 12.4 Mev–1.7 eV (Chen et al., 2021). In the *in vivo* experiments, we immobilized SD rats in a rat immobilizer, mounted the FIR device on the holder, and irradiated the rat brain with an infrared spectral emitter for 30 minutes. The rats were irradiated with FIR for 1–14 days after the MCAO. The FIR emitting device was fixed on the bracket and adjusted to 1-2 cm above the rat brain. For the *in vitro* experiments, we made homemade light-opaque boxes that could be inserted into the FIR device, and the N-2α cells (RRID: CVCL_0470) and Bend3 cells (brain-derived endothelial cells; RRID: CVCL_0170; from the US National Culture Collections) to be irradiated were placed into the box for irradiation (**Figure 1**).

The safety of FIR has been proven (Qin et al., 2024): 1. FIR is a non-contact application–Eliminating infection risks associated with implants or surgical procedures; 2. Absence of residual metabolites – Unlike pharmacological agents, FIR leaves no chemical residues *in vivo*, minimizing systemic toxicity; 3. Controllable energy delivery – Our protocol used low-intensity FIR (3.5 mW/cm^2^ at 4–14 μm wavelength).

### Rat model of middle cerebral artery occlusion

The ischemia of the brain model was established as previously detailed (Chen et al., 2022a). Anesthesia with 1.5% sodium pentobarbital (Holland) in Sprague–Dawley rats at a dosage of 40 mg/kg. The left internal, external, and bilateral carotid arteries were meticulously transected and ligated (Tu et al., 2013). Monofilament nylon (Beijing Cinontech, China) was introduced into the branching of the internal artery from the exterior artery (Chen et al., 2022).

### Assessment of neurological deficits

A rat scoring 1 point was considered to be a successful model according to the Zea Longa scoring method (Longa et al., 1989) used in the following experiments. Neurological scores were delineated as follows: 0 indicates no neurologic deficit; 1 denotes the inability to completely stretch a front paw on the affected side; 2 signifies turning towards the affected side when ambulating; 3 represents leaning towards the affected side during locomotion; and 4 reflects the incapacity to walk independently accompanied by loss of consciousness.

### Evaluation of cerebral infarct volume

Upon completion of the neurological function assessment, the rats were unconscious, and brain tissues were harvested and rinsed with a physiological saline solution (Chen et al., 2022). The tissues were positioned in a 6-cm dish and frozen at –80°C for 5–10 minutes until they were reasonably firm. The brain tissue was subsequently sectioned into six contiguous coronal slices, each approximately 2 mm in thickness, with a standard blade from the frontal pole. The sections were treated with a 2% 2,3,5-triphenyltetrazolium chloride solutions (Solarbio, Beijing, China) in an oven at 37°C for 30 minutes. Post-staining, the slices were positioned on a table with a black backdrop, displaying non-ischemic tissue in rose red and infarcted tissue in white. Images were taken with a camera that was digital and processed using ImageJ software (National Institutes of Health, Bethesda, MD, USA) (Chen et al., 2022). The volume of brain infarcts (%) was calculated as infarct area/whole brain area × 100.

### Evaluation of cerebral edema

Immediately after severing the head and removing the brain, the meninges and blood vessels were peeled off, and the left and right hemispheres were separated along the sagittal suture (operated on ice trays at an ambient temperature of 4°C, after removing the cerebrospinal fluid from the surface with a filter paper; weighed individually by using a precision electronic balance; the whole process was controlled to be completed in less than 3 minutes, to avoid the introduction of bias by evaporation of water from the tissues; the weighing was completed in less than 5 minutes after the execution.

For the swelling rate, our team used the same brain tissue wet weight difference method to assess brain edema based on a previous study (Chen et al., 2022), brain water content (%) = (left brain – right brain)/(whole brain) × 100.

### Determination of relative cerebral blood flow

Relative cerebral blood flow (rCBF) was monitored in each animal before and after the therapeutic intervention. Severe blood flow was measured 3 days post-surgery using laser diffusion flow imaging. Rats were anesthetized with 1.5% sodium pentobarbital, the scalp was incised, and the skull was exposed. The rats were placed in a PeriCam PSI laser scattering flow imager (Perimed AB, Järfälla, Sweden), with a transducer positioned 15 cm above the skull to collect images. Data was recorded for 1 minute at a sampling rate of 10 images per second. Cerebral blood flow was measured by averaging the values at each time point as a percentage of total blood flow. The ratio of the mean value at each time point to the mean value before ischemia indicated the change in rCBF.

### Data-independent acquisition (DIA) mass detection and bioinformatics analysis

Following trypsin elimination, peptides were isolated, dried using an automated centrifugal concentrator, and subsequently re-solubilized in 0.1% trifluoroacetic acid (Ren et al., 2022). Peptide quantification was conducted with a Thermo Fisher peptide quantification kit (Cat# 23275, MA, USA). Identical quantities of peptides were solubilized in mass spectrometry loading buffer and examined using liquid phase secondary mass spectrometry (LC-MS/MS; Shanghai Majorbio Technology Co., Ltd.). The accumulation and ramp periods were established at 100 ms, with a mass spectrometry scan spectrum ranging from 400–1200 m/z (Ren et al., 2022). Ion mobility values (1/K0) varied from 0.57 to 1.47 Vs·cm^2^, utilizing 64 DIA-PASEF windows (25 isolation windows). Each TIMS cycle comprises 1 MS and 10 PASEF MS/MS scans, with primary spectra charges varying from 0 to 5. The variable exclusion time for tandem mass spectrometry scans was established at 24 seconds to prevent redundant scans of parental ions, and bioinformatic analysis of proteomic data was performed utilizing the Majorbio Cloud platform (https://cloud.majorbio.com) (Ren et al., 2022). Proteomic extraction, enzymatic digestion, and quantification methods are detailed in **Additional file 1**.

The “E” in the group naming stands for “EEFIT LITE”, where it indicates the name of the FIR device we used. The unpaired t-test was employed to assess the importance of the variations across groups, with significance established at *P* < 0.05. Proteins with fold changes over 1.2 or less than 0.83 were deemed differentially expressed.

### Gene expression analysis by quantitative reverse transcription-polymerase chain reaction

Total RNA was collected from the N-2α cells utilizing the RNA kit from Favorgen Biotech (Pingdong, Taiwan, China) whereas animal tissues underwent RNA extraction with TRIzol (Invitrogen, Scotland, UK). Following extraction, the RNA content was quantified using NanoDrop Microvolume Spectrophotometers (Thermo Fisher Scientific, Waltham, MA, USA), and subsequently reverse transcribed into cDNA utilizing 10 ng of total RNA. The reverse transcription was conducted with Maxima^TM^ H Minus cDNA Synthesis Master Mix (Thermo Fisher Scientific). Following reverse transcription, the cDNA was labeled with SYBR Master Mix (Roche Diagnostics, IN, USA) and amplified with the CFX Opus Real-Time PCR System (Bio-Rad, Hercules, CA, USA). The primer sequences are listed in **Additional Table 2**. Expression of gene levels was standardized to β-actin utilizing the –2^ΔΔCt^ technique.

**Additional Table 2:** Primer sequences used for quantitative reverse transcription-polymerase chain reaction

Gene	Sequence (5’-3’)
*Plcl1*	F: AGCTGGCCTGTTTGTCTTGT R: CCCGCAAAGGGCATCATTTC
*Ncf4*	F: CCACTTTGGTGGTTGCTGAC R: CTCGATGACAAACACGGTGC
*Lgals5*	F: GACTCCATACCCGAACCTAGC R: GTGTTTCGGACCACAGCATT
*S100-A5*	F: TCCTCTCTCTTCCTTCAGAGTACA R: CCTGGTCGCTGTTTTTGTCC
*Senp3*	F: TCTCATGGCAGAGGATGGGA R: TGAGAGGAGACCCGAGTCAG
*Opa1*	F: TCTTCACTGCGGGTACACCT R: CTTCCTGTGGTGGATGCCTT
*Krt75*	F: ACTGGTGGAGCTTGAAGACG R: TGACCACAGAGATGTTCACTGG
*Omp*	F: ATTGGAATGAGGCAGACGCC R: TCGCCAAAGGTGATGAGGAAA
*Aldh2*	F: GCTGACAAGTACCACGGGAA R: CACGTTTCCAGTTGCCAAGG
*Parg*	F: TCCCTCTCAGGGTTGGTGAA R: GTGGGACCCTGAACTGTACG
*Drp1*	F: TGCAGGACGTCTTCAACACA R: GACCACACCAGTTCCTCTGG
*Mfn1*	F: GATAAAGTCCTCCCCAGCGG R: GCATGGGCCAGCTGATTAAC
*Mfn2*	F: CTCTCGATGCAACTCTATCGTC R: TCCTGTACGTGTCTTCAAGGAA
*β-actin*	F: AGCCATGTACGTAGCCATCC R: CTCTCAGCTGTGGTGGTGAA

F: Forward; Mfn: mitofusin; Opa1: optic atrophy 1; R: reverse.

### Western blotting

Cortical tissues ipsilateral to the infarction were collected on day 3 for all animal experimental groups, immediately snap-frozen in liquid nitrogen, and stored at –80°C until protein extraction. Rat cortical tissue was lysed using RIPA buffer (Cell Signaling Technology, Boston, MA, USA). Glass plates were built, and a separation gel was formulated according to the kit instructions. The separating gel was introduced, followed by a 75% alcohol layer, and allowed to rest for 30 minutes. Following the removal of the ethanol level, the highly concentrated gel was incorporated, the comb inserted, and left to stand for another 30 minutes. The comb was then removed, the electrophoresis device installed, and leakage checked. Samples (20–40 μg) were loaded, with a marker in the first lane. Electrophoresis was performed at 80 V for 30 minutes, subsequently, apply 100V until the blue bromophenol front reaches the bottom of the plate of glass plate. In the membrane transfer process, the filtration paper, gel (Epizyme Biotech, Shanghai, China), polyvinylidene fluoride membrane (Bio-Rad), and paper filter were arranged sequentially from negative to positive within the transfer folder, accompanied by the addition of pre-cooled transfer buffer. The apparatus was positioned in a 300-mA cold bath, with the transfer duration calibrated based on protein molecular weight. The polyvinylidene fluoride membrane was sectioned based on molecular weight and incubated in 5% skim milk for 1.5 hours. After blocking, the membrane underwent four washes with Tris-buffered saline with Tween 20 (TBST), each lasting 5 minutes, followed by an overnight incubation with a primary antibody solution (diluted in 3% bovine serum albumin) at 4°C. The primary antibody was retrieved, and the membrane was rinsed multiple times with TBST for 5 minutes each. The membrane was subsequently incubated with a secondary antibody solution (dilution in 3% bovine serum albumin) for 1.5 hours, followed by four washes with TBST, each lasting 5 minutes. The membrane was sprayed with enhanced chemiluminescence solution (solution A: solution B = 1:1, Invitrogen, Scotland, UK), maintained for 5 seconds, and subsequently photographed. Band intensity was measured with ImageJ software. Primary antibodies used: Opa1 (1:1000, Cat# 27733-1-AP, rat & mouse, Proteintech, Wuhan, China), 4-hydroxynonenal (4-HNE; 1:1000, Cat# A150-120A-1MG, rat & mouse, Thermo Fisher Scientific), and β-actin (1:10,000, Cat# 4967, rat & mouse, Cell Signaling Technology). Secondary antibodies used: anti-rabbit IgG, HRP-linked antibody (1:3000, Cat# 7074, rat, Cell Signaling Technology) and anti-mouse IgG, HRP-linked antibody (1:3000, Cat# 7076, mouse, Cell Signaling Technology). All primary antibody incubations were performed overnight at 4°C, while all secondary antibody incubations were conducted at room temperature for 2 hours.

### Detection of the concentration of 4-hydroxynonenal

Fresh rat serum samples were obtained and analyzed for 4-HNE concentrations utilizing a lipid oxidation 4-HNE assay kit (Abcam, Cambridge, MA, USA). The concentrations of 4-HNE were quantified utilizing a SpectraMax Paradigm Multi-Mode Microplate Reader (Molecular Devices, Sunnyvale, CA, USA) at a wavelength of 450 nm.

### Detection of the concentration of cytokines

The concentration of inflammatory cytokines was detected using LEGENDplexTM Rat Inflammatory Panel (Biolegend, San Diego, CA, USA) or LEGENDplexTM Customer Panel (Biolegend) following the manufacturer’s protocol and acquired via BD FACSAria III cell sorter (BD Biosciences, San Jose, CA, USA) (Wang et al., 2023).

### Cell culture

N-2α cells (RRID: CVCL_0470) and Bend3 cells (RRID: CVCL_0170) are from the US National Culture Collections (Thermo Fisher Scientific). Cells were grown in a standard incubator (5% CO_2_, 37°C) to achieve 60%–80% confluence for future tests. DMEM (10% FBS, 50 U/mL penicillin, and 50 µg/mL streptomycin, Gibco, MS, USA) was utilized for incubation.

### Oxygen glucose deprivation model

This work generated an *in vitro* model using the N-2α cell line subjected to OGD. The cells were categorized into three groups: control, OGD model, and OGD + FIR. On the second day, the media for the OGD model group was substituted with glucose-free DMEM (Gibco), and the cells were incubated anaerobically for 1–4 hours. In the OGD + FIR group, the media was substituted with glucose-free DMEM, and the cells were placed in an anaerobic condition (5% CO_2_, 95% N_2_, 0.1% O_2_) for 4 hours, while far-infrared radiation was administered during OGD.

### Mitochondrial DNA measurement of the number of copies

Whole genomic DNA from cellular and cerebral tissue was isolated with the FavorPrep tissue genomic DNA extraction kit (Favorgen Biotech Corp, Pingdong, Taiwan, China) and preserved at 4°C. The quantity of mitochondrial DNA (mtDNA) copies was determined utilizing quantitative reverse transcription-polymerase chain reaction (qRT-PCR) (Applied Biosystems, Sparta, NJ, USA). mtDNA copies in rats were normalized to the expression of mtDNA genes and the ND-5 gene, compared with nuclear genes and the CFTR gene. In N-2α cells, mtDNA duplication involved replacing COX2 with RSP18 in the cytoplasm, while in rats, it involved replacing CFTR with ND-5 in the cerebral cortex. The primer sequences are listed in **Additional Table 3**. The rat mtDNA copy number is the result of the correction of ND-5 in the cortex to CFTR and was calculated via the –2^ΔΔCt^ method. The mtDNA copy number is the result of the correction of COX2 in the cell to Rps18 and was calculated via the –2^ΔΔCt^ method.

**Additional Table 3:** Primer sequences used for mitochondria DNA measurement

Gene	Sequences (5’-3’)
*ND-5*	F: GGATGATGATATGGCCTTGCA R: CGACTCGGTTGTAGAGGATTGC
*CFTR*	F: AAACTCAGGATAGCTGTCCGTTTAG R: GCCAAATGATAGCATGGAACTCT
*COX2*	F: ATAACCGAGTCGTTCTGCCAAT R: TTTCAGAGCATTGGCCATAGAA
*Rps18*	F: TGTGTTAGGGGACTGGTGGACA R: CATCACCCACTTACCCCCAAAA

F: Forward; R: reverse.

### Dimethylthiazolyl-diphenyl-tetrazolium bromide assays

The cytotoxicity of FIR on our cell models was evaluated using dimethylthiazolyl-diphenyl-tetrazolium bromide (MTT) assays (5 mg/mL). N-2α cells were cultured within 96-well plates and cultured for 24 hours after wall attachment for modeling as well as FIR treatment. After treatment, 10 μL of MTT solutions (Sangon, Shanghai, China) were added and the cells were incubated in the incubator for 4–6 hours. After that, 100 μL of SDS (10% SDS dissolved in 0.01 M HCl) was added and mixed overnight. The absorbance was measured at 570 nm. Cell viability was calculated using the formula: Cell viability (%) = *A*(treated)/*A*(control) × 100.

### ATP content measurement

N-2α cells underwent FIR treatment for four hours and were subsequently rinsed with phosphate-buffered saline (PBS). A segment of PBS was pre-chilled, and the cell samples were put back in the pre-chilled PBS before being centrifuged for 5 minutes at 300 × *g* and 4°C. Two million cells were treated with 100 μL of 85% cold methanol (containing 2 μM ATP-13C10,15N5), swirled for 1 minute, and incubated on ice for ten minutes. Subsequently, the mixture was centrifuged for fifteen minutes at 13,000 × *g* and 4°C, after which the supernatant was aspirated. Seventy-five microliters of MTBSTFA and 85% methanol were added to the supernatant and vortexed for 5 minutes. The derivatized sample underwent centrifugation for 10 minutes at 13,000 × *g* and 4°C, after which 25 μL of the precipitate was fed into the LC-MS/MS apparatus (Bruker, Billerica, MA, USA) for analysis.

### Measurement of the membrane potential

In 24-well plates, 2 × 10^4^ N-2α cells per well were exposed to FIR under OGD conditions for 4 hours. Carbonyl cyanide m-chlorophenyl hydrazine (CCCP; Beyotime, Shanghai, China) at 10 mM served as a positive control. The mitochondrial membrane potential of N-2α cells stained with 5,5′,6,6′-tetrachloro-1,1′,3,3′-tetraethyl-imidacarbocyanine iodide (JC-1; Beyotime) was observed using inverted fluorescence microscopy.

### Mitochondrial permeability transition pore opening

The extent of mPTP opening in N-2α cells following OGD and FIR therapy was assessed utilizing the mPTP Fluorescence Detection Kit (Beyotime). Following two washes of the cells with pre-warmed PBS, the cells were stained with Calcein AM, and the staining solution was then replaced with standard DMEM media (Agilent, Santa Clara, CA, USA) after 40 minutes in the cell incubator. They were thereafter incubated in a standard incubator shielded from light. The cells were subsequently rinsed twice with PBS and examined using an inverted fluorescence microscope (Nikon ECLIPSE 80i, Tokyo, Japan).

### Seahorse XF Mito stress test assay

Cells were plated in XFp Cell Culture Miniplates (Agilent, Santa Clara, CA, USA) at 4000 cells/well. After 24 hours, mitochondrial respiration was determined with XFp Cells Mito Stress Test Kit (Agilent) on Seahorse Bioscience XFp extracellular flux analyzer (Agilent). The mitochondrial oxygen consumption rate (OCR) was measured by serial injection of oligomycin (10 μmol), carbonyl cyanide 4-(trifluoromethoxy) phenylhydrazone (FCCP) (0.5 μmol), and 0.5 μmol mix of rotenone (complex I inhibitor) and antimycin A (complex III inhibitor). Data analysis was performed with Seahorse Wave Pro software (Agilent).

### MitoTracker Green staining

N-2α cells were treated with MitoTracker Green (Beyotime) for twenty minutes at a final dosage of 100 nM Hoechst 33342 (Beyotime) to examine mitochondrial morphology. Following the removal of the supernatant, the cells were analyzed with a Leica TCS SP5 confocal microscope (Leica Microsystems, Wetzlar, Germany). Each point in the scatterplot represents a single cell measurement, and statistical differences were calculated based on at least 18 data points from 3 independent experiments (*n* = 3).

### Transmission electron microscopy

N-2α cells were collected and immersed in electron microscopy fixing agent (Servicebio, Wuhan, China) at 4°C. The specimens were subsequently preserved in precooled fixative and dispatched to Servicebio for additional processing and sectioning. To observe the ultrastructural changes of neuronal mitochondria in the cerebral cortex, the cerebral cortex was immobilized in an electron microscopy fixing agent after sampling, and the subsequent steps were consistent with the *in vitro* experimental operations. Images were acquired with a transmission electron microscope (HT7800, HITACHI, Japan). Quantitative indicators for transmission electron microscopy (TEM) include perinuclear mitochondrial area and mitochondrial circularity index in N-2α cells.

### Small interfering RNA transfection and MYLS22 inhibitor administration

The Opa1-small interfering RNA (siRNA), recombinant mitofusin (Mfn)1/2-siRNA, and negative control-siRNA were provided by Sangon. N-2α cells were used using Lipofectamine® RNAiMAX (Invitrogen) according to the manufacturer’s instructions. 24 hours after siRNA treatment, cells were incubated under OGD conditions and treated with FIR as mentioned above, then all cells were harvested for further experiments. For pharmacological inhibition, MYLS22 (MedChemExpress, NJ, USA) was added 24 hours pre-OGD. At 24 hours post-administration, all groups underwent OGD with/without FIR treatment before harvest.

### Angiogenesis experiment

Matrigel gel (15 µL, Corning, NY, USA) was aspirated and slowly dispensed vertically into the center of a well in a 24-well plate. Using a pre-chilled 1 mL pipette tip, the Matrigel was gently spread in a circular motion to cover the central area. We used a 10 µL pipette tip to carefully apply the Matrigel along the periphery of the well. This avoided touching the edges and ensured complete, even coverage across the entire bottom surface. The coated plate was placed on a level surface and left undisturbed for 10 minutes at room temperature. It was then transferred to a 37°C incubator for 30 minutes to allow complete polymerization of the Matrigel. Following solidification, the gel was hydrated by adding DMEM medium to the well. PBS was added to the inter-well spaces to maintain humidity. Between 80,000 and 150,000 Bend.3 cells were seeded onto the hydrated Matrigel surface. The cell suspension was dispensed dropwise into the well using a vertically held pipette tip, allowing it to distribute naturally. The plate was then gently swirled to ensure even cell distribution without disrupting the gel, before being returned to the 37°C incubator. Tube formation was monitored under a microscope after 4–6 hours of incubation. A 1× working solution of Calcein-AM (Beyotime, Shanghai, China) was prepared from the 1000× stock. Subsequently, 150–200 µL of the Calcein-AM solution was added to each well, followed by incubation at 37°C for 15–25 minutes. Images were acquired using a fluorescence microscope. Quantitative analysis of tube formation was performed using ImageJ software to assess the following parameters: number of master segments, total master segment length, number of meshes, and number of master junctions.

### Gene Ontolog and Kyoto Encyclopedia of Genes and Genomes pathway analyses

We performed the Gene Ontology (GO) annotation and pathway enrichment analysis utilizing the Kyoto Encyclopedia of Genes and Genomes (KEGG), specifying the species as “Rattus norvegicus sapiens.” Three categories were examined in the GO analysis: molecular function (MF), cellular component (CC), and biological process (BP). The graph was produced utilizing Bioinformatics (www.bioinformatics.com.cn), a complimentary web free online platform for bioinformatics data analysis.

### Statistical analysis

Statistical analyses were performed with GraphPad Prism 8.0 software. Results are expressed as mean ± standard error of the mean (SEM). Differences were significant if the value of *P* was below 0.05. Comparisons between groups for data with a normal distribution were conducted using a unpaired *t*-test or a one-way analysis of variance. The rank sum test and Kruskal-Wallis test were employed for non-normally distributed data.

## Results

### Far-infrared radiation treatment reduces neurological deficits and infarct volumes in cerebral ischemia rats

**Figure 2:**
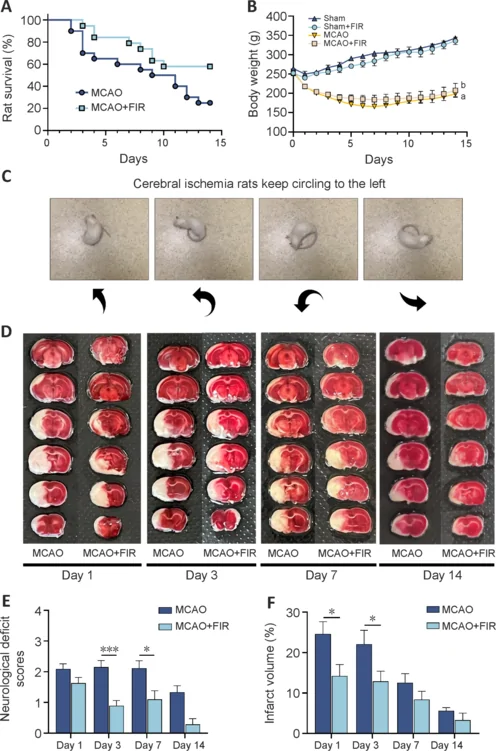
FIR therapy mitigates ischemic brain injury in MCAO rats. (A) Survival rates of MCAO and MCAO + FIR groups over 14 days post-surgery (*n* = 18). (B) Body weight changes in rats from each group post-FIR irradiation compared with non-FIR irradiation (*n* ≥ 3). Sham *vs.* MCAO: ^a^*P* < 0.0001; Sham + FIR *vs.* MCAO + FIR: ^b^*P* < 0.0001. (C) Behavioral changes in SD rats following MCAO. (D) Representative photos of coronal brain infarct slices stained with TTC, illustrating infarct volume (*n* ≥ 7). White indicates infarct regions. (E) Longa neurological deficit scores in FIR-irradiated groups compared with non-FIR-irradiated groups (*n* ≥ 6). (F) Infarct volume in FIR-irradiated groups *versus* non-FIR-irradiated groups (*n* ≥ 7). The data are expressed as the mean ± SEM. *P < 0.05, ***P < 0.001. FIR: Far-infrared radiation; MCAO: middle cerebral artery occlusion; TTC: 2,3,5-triphenyltetrazolium chloride.

To evaluate the neuroprotective effects of FIR against cerebral ischemia, we analyzed survival rates, infarct volume, neurological deficits, and cerebral edema in a MCAO model. Tissue samples were collected sequentially from postoperative day 2 onward. Over a 14-day observation period, FIR irradiation improved survival rates from 25% in untreated MCAO rats to 60% in FIR-treated animals (**Figure 2A**). Body weight remained comparable between FIR-treated and untreated MCAO groups across all time points (**Figure 2B**), confirming that FIR did not adversely affect normal physiological development.

Neurological impairment assessed using a standardized scoring system based on motor asymmetry and rotational behavior (Longa et al., 1989), revealed severe deficits in MCAO rats, characterized by persistent counterclockwise circling (**Figure 2C**). MCAO-induced cerebral injury manifested as distinct ischemic infarcts visible in brain tissue sections. FIR treatment consistently reduced infarct volume compared with untreated MCAO controls (**Figure 2D**). And, FIR treatment also markedly reduced neurological scores at all evaluated time points (days 1, 3, 7, and 14 post-ischemia, **Figure 2E**).

Quantitative analysis demonstrated reductions in infarct size at early time points: FIR decreased infarct volume by 42% on day 1 (24.64% *vs.* 14.22%, *P* < 0.05) and 41% on day 3 (22.12% *vs.* 12.89%, *P* < 0.05). This protective effect persisted through days 7 and 14, but no statistically significant difference was seen, with further volumetric improvements observed in FIR-treated rats (**Figure 2F**).

### Comparison of the therapeutic effects of far-infrared radiation irradiation with those of nimodipine in cerebral ischemia rats

**Figure 3:**
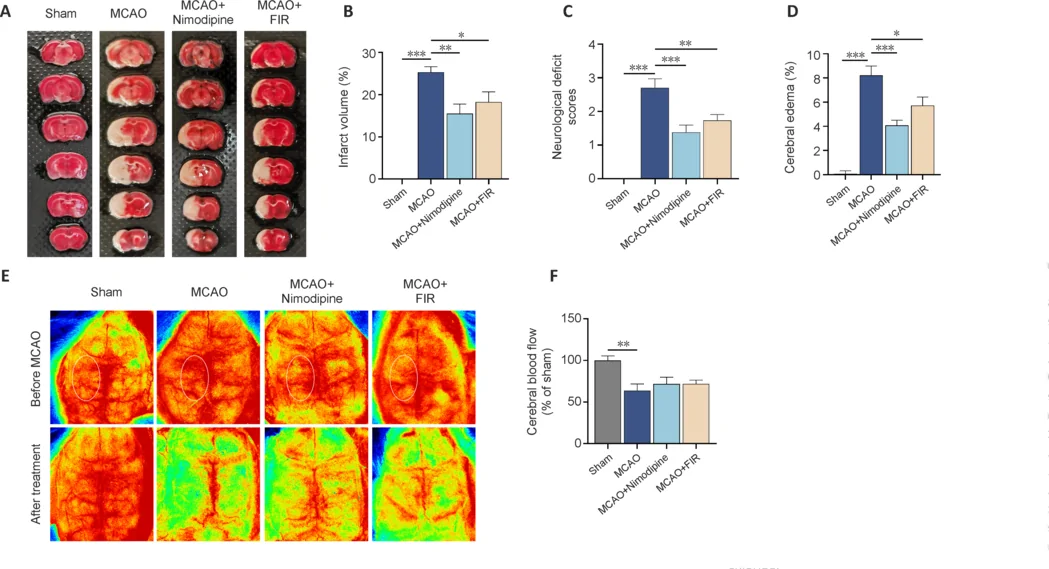
Therapeutic effects of FIR treatment in a rat model of cerebral ischemia. (A) Representative TTC-stained sagittal brain sections (*n* ≥ 8). White indicates infarct regions. (B) Quantitative analysis of infarct volume in MCAO rats (*n* ≥ 8). (C) Longa neurological deficit scores in the sham, model, FIR, and nimodipine groups of rats in MCAO condition (*n* ≥ 18). (D) The cerebral edema in the sham, model, FIR, and nimodipine groups of rats in MCAO condition (*n* ≥ 7). (E, F) Laser speckle contrast images depicting rCBF in FIR-treated MCAO rats, accompanied by statistical analysis of rCBF (*n* ≥ 7). The data are expressed as the mean ± SEM. *P < 0.05, **P < 0.01, ***P < 0.001. FIR: Far-infrared radiation; MCAO: middle cerebral artery occlusion; rCBF: relative cerebral blood flow; TTC: 2,3,5-triphenyltetrazolium chloride.

FIR therapy, a novel treatment modality for cerebral ischemia, warrants comparison with established clinical treatments. Nimodipine, a calcium antagonist, is commonly used for neuronal protection after cerebral ischemia and for the treatment of vascular dementia (Salvadori et al., 2021; Yang et al., 2022b, 2024; Wei et al., 2023). In our study, the efficacy of FIR irradiation in treating cerebral ischemia was assessed in comparison with nimodipine, which served as the positive control. Results indicated that both FIR irradiation and nimodipine markedly suppressed neurological deficit scores and cerebral infarct volume and decreased the rate of cerebral edema (**Figure 3A–D**). Notably, the nimodipine treatment group exhibited more pronounced reductions in behavioral scores, infarct volume, and cerebral edema compared with the FIR treatment group. Furthermore, the rCBF was also evaluated in the cortex of experimental rats before and after ischemia. As shown in **Figure 3E**, pre-MCAO imaging revealed full blood flow (bright red color) in all groups. However, distinct yellowish-green ischemic areas were observed in all the model groups after MCAO surgery, indicating varying degrees of ischemia in the brain region of MCAO rats. In contrast, the rCBF recovery values in the MCAO+FIR group were higher than those in the MCAO group, suggesting that FIR partially improved cerebral blood flow. However, there was no statistical difference between the 2 groups because of large individual differences (**Figure 3F**). Nimodipine and FIR treatment groups presented partial CBF recovery without a statistically significant difference.

We observed in our *in vitro* experiments that FIR indeed induced angiogenesis and that FIR significantly increased the tube-forming capacity of Bend3 cells (**Additional Figure 1A** and **B**). This also helped us to confirm the conclusion that FIR has the potential to help blood flow recover after stroke. Our data indicate that FIR irradiation therapy exerts a protective impact on the cerebral blood flow of ischemic rats. However, its efficacy is inferior to that of nimodipine.

### Proteomic analysis of ischemic brain tissue from middle cerebral artery occlusion rats

**Figure 4:**
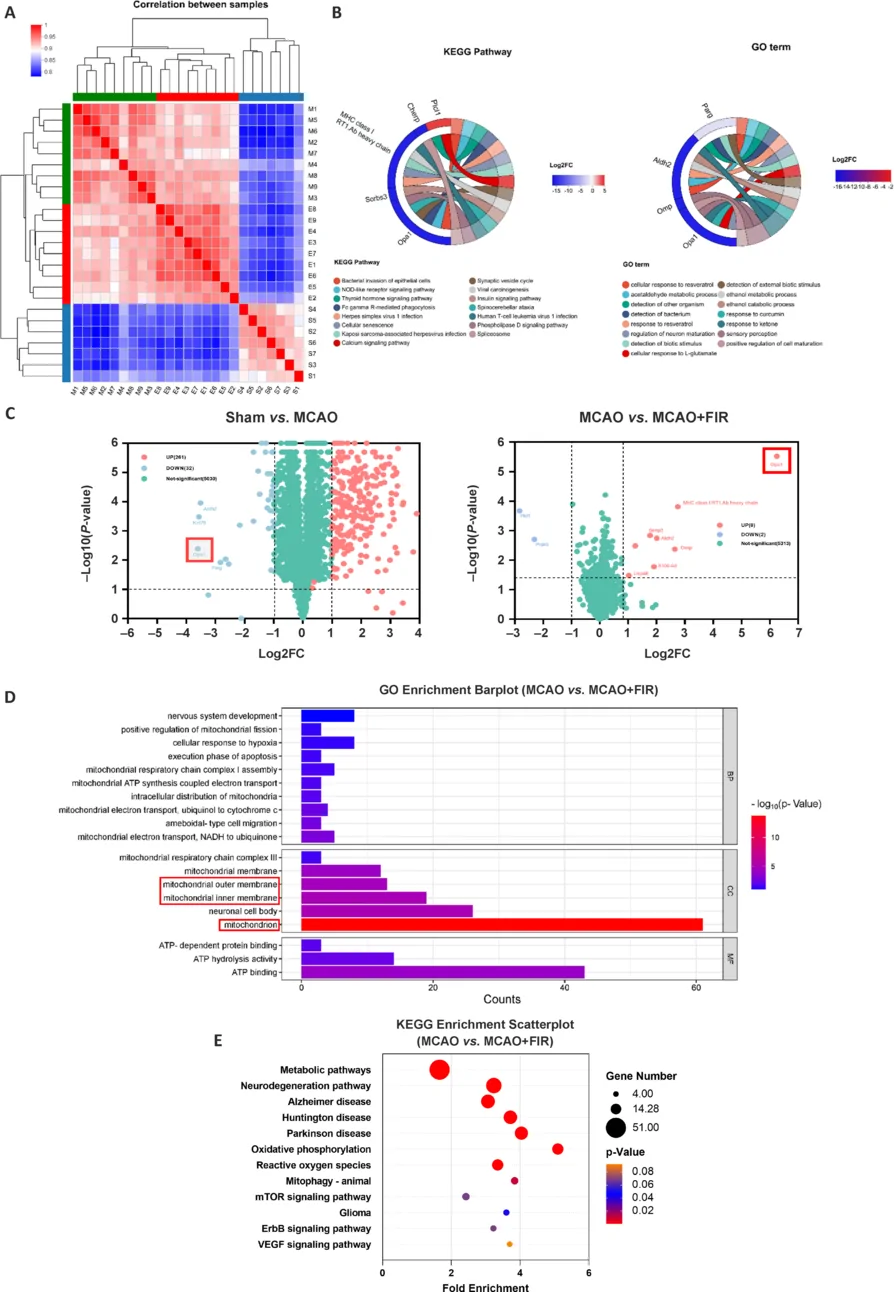
Proteomic profiling of ischemic brain tissue in MCAO rats reveals FIR-mediated molecular modulation. (A) Hierarchical clustering heatmap of protein expression profiles across sham, MCAO, and FIR-treated groups. Columns represent samples; rows depict protein clustering. S# (Sham group): where only carotid vessel exposure was performed but no wire bolt was inserted as surgical control; M# (MCAO group): middle cerebral artery occlusion modeling group (Middle Cerebral Artery Occlusion), underwent standard MCAO surgery; E# (MCAO + FIR group): MCAO combined with FIR group, receiving FIR intervention based on the MCAO model. (B) KEGG pathway (left) and GO term (right) chord diagrams highlighting enriched pathways and biological functions in MCAO *versus* FIR-treated groups. (C) Volcano plot illustrates the fold change of all gene datasets from sham, MCAO, and FIR treatment groups. (D) GO term diagram: BP represents biological processes, CC represents cellular components, and MF represents molecular functions (MCAO *vs.* MCAO + FIR). (E) Bubble diagram of KEGG enrichment items (MCAO *vs*. MCAO + FIR). The X-axis denotes the enrichment factor, whereas the Y-axis signifies KEGG keywords (*n* ≥ 7). FIR: Far-infrared radiation; GO: Gene Ontology; KEGG: Kyoto Encyclopedia of Genes and Genomes; MCAO: middle cerebral artery occlusion.

To investigate the molecular mechanisms underlying FIR therapy in cerebral ischemia-reperfusion injury, we performed a proteomic analysis of brain tissues from FIR-treated MCAO rats. Hierarchical clustering of protein expression profiles revealed distinct differences between the sham, MCAO, and FIR-treated groups, demonstrating divergence in protein expression patterns (**Figure 4A**). Notably, FIR therapy changed the expression changes of differentially expressed proteins (DEPs) observed in MCAO rats.

To further characterize these molecular changes, KEGG and GO chord diagram analyses were conducted. These analyses identified Opa1, a protein downregulated in the MCAO group, as a key player in pathways linked to neuronal regulation, including positive regulation of response to stimulus, neuron maturation, and the NOD-like receptor signaling pathway (**Figure 4B**). Differential expression analysis (*P*-adjust < 0.05, |log_2_FC| ≥2) revealed 5,322 DEPs in MCAO rats. Strikingly, FIR treatment changed the expression of 10 DEPs (8 upregulated, 2 downregulated) in brain tissues compared with the untreated MCAO group (**Figure 4C**). As shown in **Figure 4C**, Opa1 protein expression was significantly downregulated in the brain tissues of rats in the MCAO group (*P* < 0.01, *vs.* the Sham group), whereas Opa1 levels were significantly restored after FIR intervention (MCAO + FIR group) compared with the MCAO group (*P* < 0.05). This dynamic change was further validated in the volcano map of differentially expressed genes - the upregulation trend of Opa1, a key protein, was in the region of significant difference in the FIR-treated group (indicated by the red box).

GO functional classification categorized these unigenes into three major domains: MF, BP, and CC (**Figure 4D**). Enriched terms included nervous system development (BP), mitochondria (CC), and protein binding (MF), underscoring the potential role of FIR in neuronal repair and mitochondrial regulation. Complementary KEGG pathway analysis highlighted enrichment of DEPs in 12 pathways, several of which are implicated in neurological disorders (**Figure 4E**). **Figure 4E** showed that several neurodegenerative disease-related pathways were significantly enriched, with Huntington’s disease and mitochondrial autophagy pathway genes being the most significantly enriched, suggesting that FIR may alleviate neurological damage through mitochondria. In addition, the enrichment of the oxidative phosphorylation pathway indicated that MCAO induced mitochondrial energy metabolism disorders, and the potential association between the mTOR signaling pathway and reactive oxygen species pathway further supported the involvement of oxidative stress. These results suggested that FIR intervention might exert neuroprotective effects through restoring mitochondrial function, inhibiting oxidative stress, and regulating energy metabolism.

Ten critical proteins were identified based on a fold change of more than 2 or less than 0.5, as seen in **Additional Figure 2A**. Additionally, the expression levels of the hub genes in the MCAO and FIR treatment groups were confirmed by qRT-PCR. The findings indicated that the mRNA levels of mitochondrial genes, including Opa1, Senp3, and aldehyde dehydrogenase 2 (Aldh2), were higher in the FIR treatment group compared with the MCAO group (**Additional Figure 2B**). The findings indicate a potential role of mitochondrial function in FIR therapy.

### FIR treatment protects against ischemic damage to the brain in middle cerebral artery occlusion rats via the upregulation of mitochondrial-associated proteins

**Figure 5:**
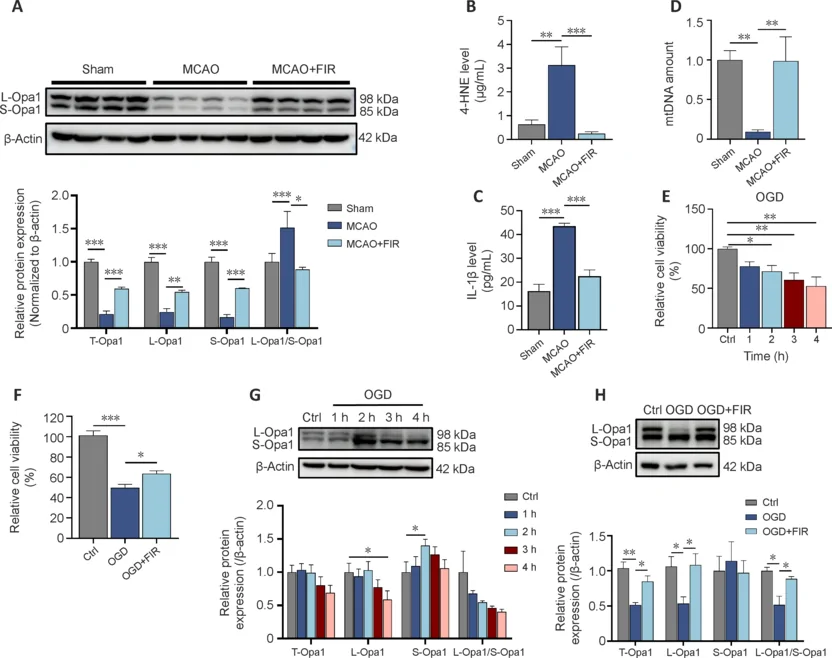
FIR treatment protects against brain injury by inducing the gene expression of mitochondrial dynamics proteins. (A) Western blot images and quantitative analysis of Opa1 protein expression in brain tissue of MCAO rats treated with or without FIR (*n* = 4). (B, C) Serum 4-HNE (B) and IL-1β levels (C) in MCAO rats treated with or without FIR (*n* ≥ 5 for 4-HNE and *n* = 6 for IL-1β). (D) Quantitative analysis of mtDNA levels in MCAO rats with and without FIR treatment (*n* ≥ 7). (E) Cytotoxic effects of OGD in N-2α cells assessed by MTT assay. (F) FIR treatment mitigated the OGD-induced cytotoxicity in N-2α cells (*n* = 3). (G) Western blot images and quantitative analysis of Opa1 expression in N-2α cells after 1–4 hours of OGD treatment (*n* = 3). (H) Western blot images and quantitative analysis of Opa1 protein expression in N-2α cells with and without FIR after 4 hours of OGD treatment (*n* = 3). The data are expressed as the mean ± SEM. *P < 0.05, **P < 0.01, ***P < 0.001. 4-HNE: 4-Hydroxynonenal; Ctrl: control; FIR: far-infrared radiation; IL: interleukin; L-Opa1: membrane-anchored long form of Opa1; MCAO: middle cerebral artery occlusion; mtDNA: mitochondrial DNA; MTT: dimethylthiazolyl-diphenyl-tetrazolium bromide; OGD: oxygen-glucose deprivation; Opa1: optic atrophy protein 1; S-Opa1: processed short form of Opa1; T-Opa1: total Opa1.

Following proteomic analysis, we investigated the impact of FIR on mitochondrial-associated proteins *in vivo*. Optic atrophy protein 1 (Opa1), a critical mediator of mitochondrial fusion in neurons, exists in two isoforms: the membrane-anchored long form (L-Opa1) and the processed short form (S-Opa1). While L-Opa1 promotes mitochondrial fusion, excess S-Opa1 drives mitochondrial fission, and an imbalance between these isoforms leads to mitochondrial fragmentation and dysfunction (Zhang et al., 2023; Fry et al., 2024; Shadman et al., 2024). In the MCAO group, total Opa1 (T-Opa1) and L-Opa1 levels were reduced compared with sham controls (**Figure 5A**). FIR treatment significantly restored T-Opa1 and L-Opa1 expression in MCAO rats (*P* < 0.05), suggesting a role in rebalancing mitochondrial dynamics.

To evaluate mitochondrial oxidative stress, serum levels of 4-HNE, a lipid peroxidation marker, were analyzed (Guo et al., 2013; Xia et al., 2020). MCAO rats exhibited elevated 4-HNE levels compared with sham controls, which FIR treatment markedly suppressed (**Figure 5B**). Beyond mitigating oxidative stress, FIR therapy may demonstrate systemic anti-inflammatory effects in MCAO rats. Quantification of serum cytokines at 72 hours post-ischemia revealed FIR suppressed IL-1β release (**Figure 5C**). Furthermore, mtDNA copy number, a marker of mitochondrial integrity, was reduced in the infarcted cortex of MCAO rats but recovered post-FIR treatment (**Figure 5D**). These findings collectively indicate that FIR alleviates ischemia-induced oxidative stress and mitochondrial damage.

Using N-2α cells subjected to OGD, we assessed the protective effects of FIR. OGD reduced cell viability by **~**50% at 4 hours (**Figure 5E**), while FIR irradiation restored survival rates (**Figure 5F**). OGD also decreased T-Opa1 and L-Opa1 protein levels in a time-dependent manner, concurrent with a rise in S-Opa1 (**Figure 5G**), consistent with excessive mitochondrial fission (Anand et al., 2014; Ge et al., 2020). FIR treatment improved these effects, restoring T-Opa1 and L-Opa1 expression while partially normalizing S-Opa1 levels (**Figure 5H**), thereby suggesting that it is possible that FIR can maintain homeostasis in this dynamic process of mitochondrial dynamics (Song et al., 2009). These results demonstrate FIR’s capacity to counteract OGD-induced mitochondrial fragmentation and cell death.

### Far-infrared radiation treatment improves mitochondrial function in N-2α cells after oxygen-glucose deprivation-mediated injury

**Figure 6:**
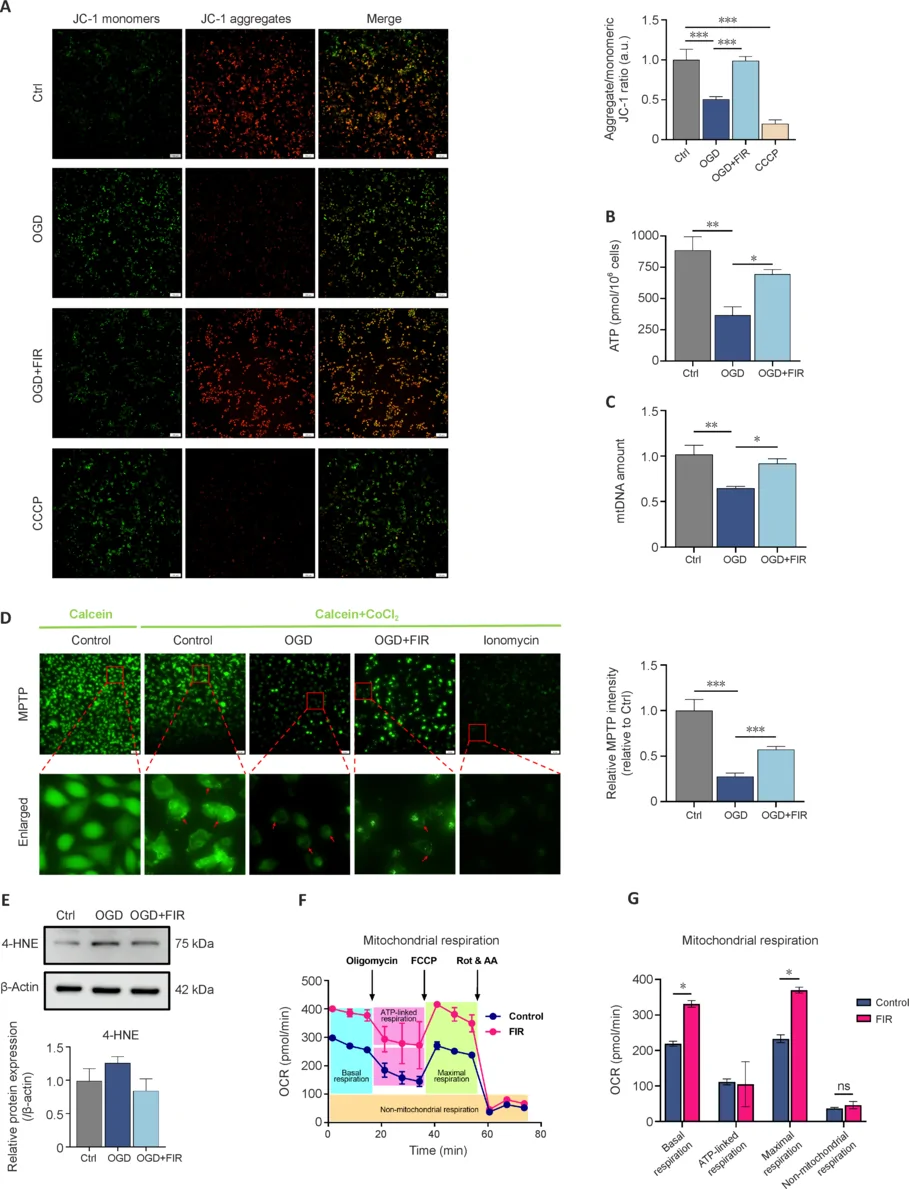
FIR therapy restores mitochondrial function in OGD-injured N-2α cells. (A) Images of JC-1 staining in OGD-treated N-2α cells, both with and without FIR treatment. CCCP functioned as a positive control on the dissipation of mitochondrial membrane potential. Bar charts provide the quantitative assessment of the red/green fluorescence intensity ratio. (B) ATP production (pmol/10^6^ cells) in OGD-treated N-2α cells, with and without FIR treatment. (C) Quantification of mtDNA by quantitative reverse transcription-polymerase chain reaction in OGD-treated N-2α cells post-FIR treatment. (D) Degree of MPTP opening in OGD-treated N-2α cells, with and without FIR treatment. N-2α cells were treated for 30 minutes in Calcein AM (1X) staining solution, CoCl2 solution, or fluorescence quenching solution (Ionomycin). Calcein AM is sequestered within the cell, producing intense green fluorescence. CoCl_2_ is unable to traverse the mitochondrial membrane. Co-loading with CoCl_2_ diminishes calcein-induced fluorescence in the cellular matrix while preserving mitochondrial fluorescence. The activation of MPTP by Ionomycin facilitates the entry of CoCl_2_ into mitochondria, resulting in the attenuation of mitochondrial green fluorescence. Bar charts show the quantitative analysis of the fluorescence signal. (E) Western blot images and quantitative analysis of 4-HNE protein expression in OGD-treated N-2α cells post-FIR treatment. Bar charts show the statistical analysis of relative 4-HNE protein expression. (F) The mitochondrial respiration profile of untreated cells and FIR-treated cells. (G) The mitochondrial respiration test determines the key parameters of mitochondrial function by directly measuring the OCR of cells. The compounds (oligomycin, FCCP, and a mix of rotenone and antimycin A) were serially injected to measure the basal respiration, ATP-linked respiration, and maximal respiration, respectively. Data are expressed as the mean ± SEM (*n* = 3). *P < 0.05, **P < 0.01, ***P < 0.001. 4-HNE: 4-Hydroxynonenal; CCCP: carbonyl cyanide m-chlorophenyl hydrazine; Ctrl: control; FIR: far-infrared radiation; JC-1: 5,5′,6,6′-tetrachloro-1,1′,3,3′-tetraethyl-imidacarbocyanine iodide; MCAO: middle cerebral artery occlusion; mPTP: mitochondrial permeability transition pore; mtDNA: mitochondrial DNA; ns: not significant; OCR: oxygen consumption rate; OGD: oxygen-glucose deprivation.

Given the established role of far-infrared radiation in promoting mitochondrial fusion and inhibiting fission—processes critical to mitochondrial function—we investigated whether FIR therapy preserves mitochondrial integrity by modulating the mPTP and associated parameters. The mPTP, a key mediator of mitochondrial membrane potential (∆Ψm) collapse, is linked to mitochondrial dysfunction and irreversible damage (Yu et al., 2022). To assess ∆Ψm, we employed JC-1, a fluorescent probe that fluoresces red (aggregated form, high ∆Ψm) or green (monomeric form, low ∆Ψm) depending on mitochondrial health (Sivandzade et al., 2019). FIR treatment markedly attenuated OGD-induced ∆Ψm loss in N-2α cells, correlating with reduced early apoptosis (**Figure 6A**).

FIR therapy also restored mitochondrial bioenergetics. LC‒MS/MS analysis revealed that FIR improved the OGD-driven decline in ATP production (**Figure 6B**), while qPCR demonstrated that FIR rescued mtDNA copy numbers suppressed by OGD (**Figure 6C**). Furthermore, calcein-AM retention assays showed that FIR inhibited OGD-induced mPTP opening, preserving mitochondrial integrity (**Figure 6D**). These findings collectively highlight FIR’s ability to sustain mitochondrial homeostasis under ischemic stress.

Consistent with *in vivo* observations, FIR partially reduced 4-HNE levels in OGD-challenged cells, but there was no statistical difference (**Figure 6E**). In addition, we next measured whether FIR elevated mitochondrial respiratory capacity in cells using the Seahorse XF method. As shown in **Figure 6F**, the peak was elevated in the FIR group compared with the Control group, suggesting that FIR can increase mitochondrial respiratory capacity. Our results reveal that FIR treatment markedly increases basal mitochondrial respiration (*P* < 0.05) and maximal oxygen consumption rate (OCR, *P* < 0.05) relative to the control group, as assessed via Seahorse XF metabolic flux analysis (**Figure 6G**). These findings suggest that FIR is associated with improved mitochondrial energy metabolism function. Together, these results demonstrate that FIR therapy enhances mitochondrial resilience by restoring ATP synthesis, maintaining ∆Ψm, stabilizing mtDNA, blocking mPTP opening, mitigating oxidative stress, and OCR analysis (**Figure 6A–G**).

### Far-infrared radiation promotes cellular mitochondrial fusion and restores cellular mitochondrial morphology

**Figure 7:**
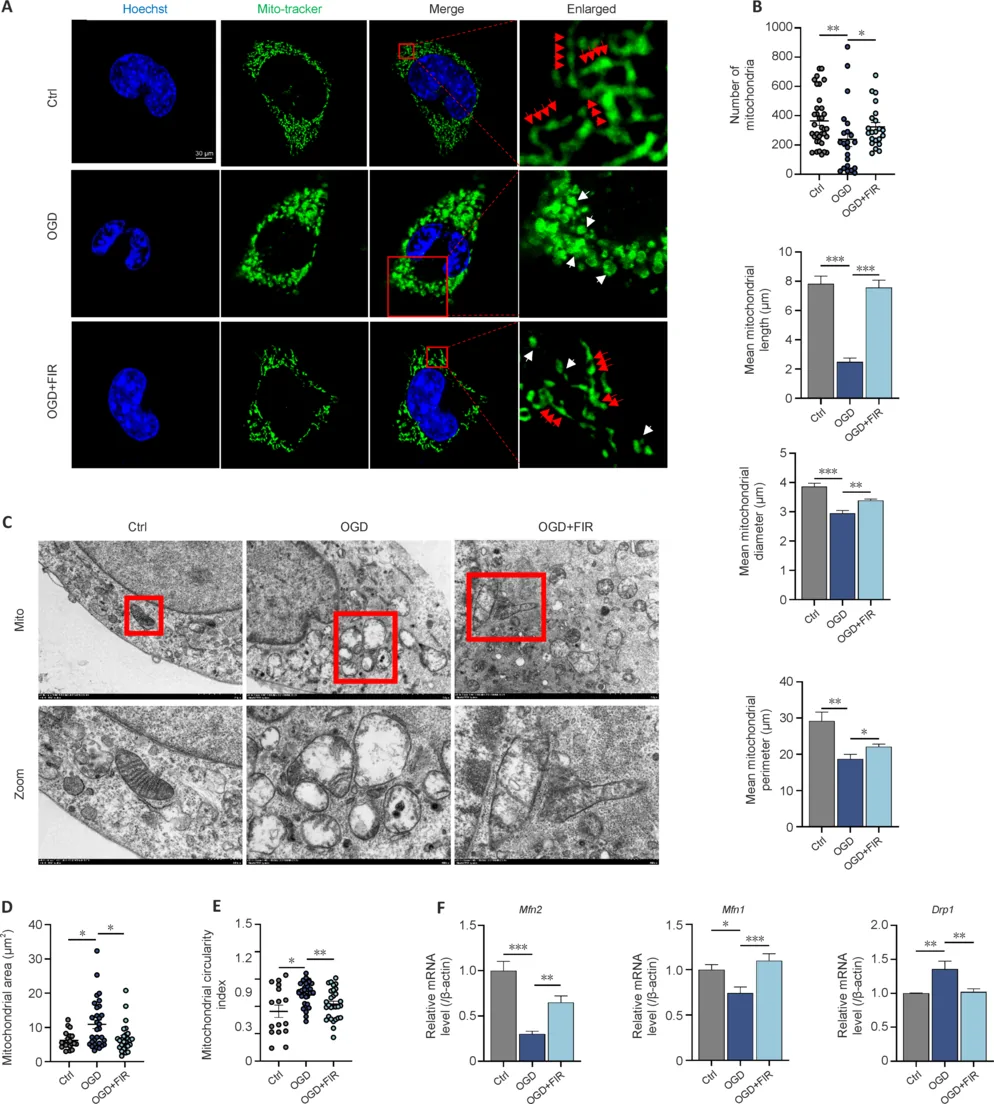
FIR promotes cellular mitochondrial fusion and restores cellular mitochondrial morphology. (A) Mitochondria in N-2α cells were labeled with Mito Tracker Green (original magnification 63×), and mitochondrial morphology was analyzed using laser scanning confocal microscopy. (B) Quantification of mitochondrial number, length, diameter, and perimeter in N-2α cells. (C) Transmission electron microscopy images showing the impact of FIR treatment on OGD-treated N-2α cells. (D, E) Measurement of perinuclear mitochondrial area and mitochondrial circularity index in N-2α cells. (F) *Mfn2*, *Mfn1*, *Drp1* gene expression related to mitochondrial dynamics in OGD-treated N-2α cells post-FIR treatment using quantitative reverse transcription-polymerase chain reaction. Data are expressed as the mean ± SEM (*n* = 3). *P < 0.05, **P < 0.01, ***P < 0.001. Ctrl: Control; FIR: far-infrared radiation; Mfn: mitofusin; OGD: oxygen-glucose deprivation.

Building on evidence that FIR enhances mitochondrial function and mitigates damage, we explored its role in modulating mitochondrial fusion via Opa1 upregulation. Mitochondrial fusion is essential for maintaining structural integrity, which we assessed using MitoTracker Green staining. Under normal conditions, mitochondria exhibited elongated, interconnected networks (**Figure 7A**). OGD, however, induced severe fragmentation, disrupting mitochondrial architecture. Quantitative analysis revealed reductions in mitochondrial number, size (average area and diameter), and perimeter in OGD-challenged cells, all of which were restored by FIR treatment (**Figure 7B**).

TEM further corroborated these findings: OGD caused mitochondrial swelling, cristae loss, and formation of spherical organelles, whereas FIR preserved cristae density and ultrastructural integrity (**Figure 7C**). Opa1, a key regulator of cristae remodeling (Frezza et al., 2006; An et al., 2013), was downregulated by OGD but rescued by FIR. This restoration of Opa1 expression correlated with attenuated fragmentation and partial recovery of mitochondrial morphology (**Figure 7D** and **E**).

To dissect the impact of FIR on mitochondrial dynamics, we analyzed the expression of fusion- (*Mfn1*, *Mfn2*) and fission-related (*Drp1*) genes. FIR improved OGD-driven suppression of Mfn1 and Mfn2 while inhibiting Drp1 upregulation (**Figure 7F**), shifting the balance toward fusion. FIR may modestly modulate Mfn1/2 mRNA expression as a secondary consequence of Opa1 activation, but functional evidence confirms Mfn1/2 are dispensable for the core protective mechanism of FIR (**Additional Figure 3A**). These results underscore the ability of FIR to stabilize mitochondrial dynamics, thereby preserving bioenergetic function and structural homeostasis.

### Far-infrared radiation promotes mitochondrial morphology and exhibits a protective effect on N-2α cells via activation of Opa1

**Figure 8:**
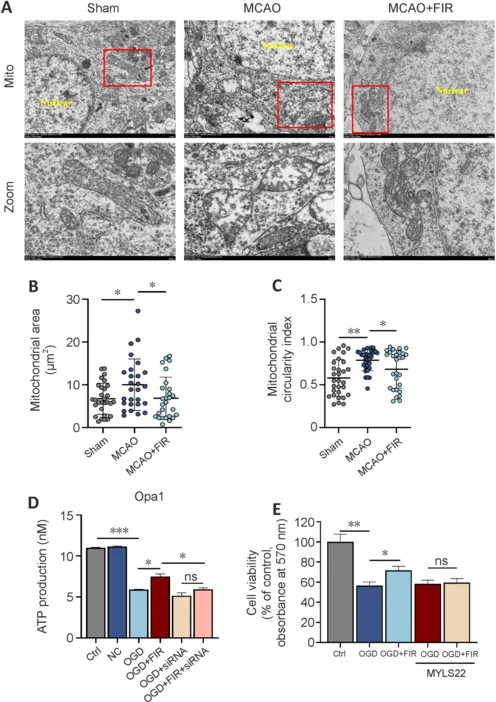
FIR promotes mitochondrial morphology and exhibits a protective effect on N-2α cells via activation of Opa1. (A) Representative TEM images revealing mitochondrial morphological and structural changes in the MCAO rat brain cortex. Magnified images are from red boxes. (B, C) Quantitative analysis of mitochondrial area and mitochondrial circularity index based on electron microscope images of MCAO rat mitochondria. (D) ATP production (nM) in OGD-treated N-2α cells in each group. (E) Cell viability after FIR treatment after 24 hours of MYLS22-treated N-2α cells. The data are expressed as the mean ± SEM (*n* = 3). *P < 0.05, **P < 0.01, ***P < 0.001. Ctrl: Control; FIR: far-infrared radiation; MCAO: middle cerebral artery occlusion; ns: not significant; OGD: oxygen-glucose deprivation; Opa1: optic atrophy 1.

Critically, FIR-restored mitochondria architecture strongly correlated with Opa1 levels (Varanita et al., 2015; Pohl et al., 2023), mechanistically explaining its bioenergetic rescue effects. TEM imaging provided evidence demonstrating that FIR treatment protects mitochondrial structure and function *in vitro*. We next investigated whether FIR preserves mitochondrial ultrastructural and functional integrity in the ischemic cortex of MCAO rats using TEM *in vivo*. As shown in **Figure 8A**, mitochondria in sham group exhibited intact ultrastructural architecture, featuring continuous inner/outer membranes, densely packed cristae, and electron-dense matrices. Conversely, the MCAO rat displayed pathological mitochondrial swelling, cristae fragmentation, and matrix vacuolization. FIR treatment partly mitigated the observed damage to mitochondrial structures. Quantitative image analysis showed increased mitochondrial area in the cerebral cortex of MCAO rats (**Figure 8B**) and increased circularity index (**Figure 8C**). Mitochondria from FIR-treated MCAO rats partially reversed these outcomes. Results from *in vivo* experiments indicated that FIR effectively alleviated mitochondrial damage induced by stroke.

To determine whether the effects of FIR depend on Opa1, we used specific siRNA to knock down Opa1 expression *in vitro*, and transfection with 20 nM and 50 nM siRNAs found that they were equally effective in knocking down Opa1 expression, and then followed up with 20 nM Opa1-siRNA (**Additional Figure 3B**). The transfection with 20 nM Opa1-siRNA was followed by transfection with 20 nM Opa1-siRNA. Subsequent FIR treatment no longer up-regulated Opa1 mRNA levels (**Additional Figure 3C**). The sequences of the three siRNAs and NC-siRNA are shown in **Additional Table 4**. All siRNA sequences were designed by Sangon.

**Additional Table 4:** siRNA sequence

Name		Sequences (5’-3’)
Opal-siRNA	Sense Antisense	CAGCAGUUCUCUUCUCUAA UUAGAGAAGAGAACUGCUG
Mfnl-siRNA	Sense Antisense	GCAAGAGCUCUGUCAUCAA UUGAUGACAGAGCUCUUGC
Mfn2-siRNA	Sense Antisense	GGAAGAGCACCGUGAUCAA UUGAUCACGGUGCUCUUCC
Negative control-siRNA	Sense Antisense	UUCUCCGAACGUGUCACGUTT ACGUGACACGUUCGGAGAATT

Meanwhile, we directly detected ATP production to observe whether FIR therapy rescues mitochondrial damage directly through Opa1. Using the ATP assay kit, we found that FIR was not effective in rescuing mitochondrial dysfunction (via ATP release) after Opa1-siRNA-mediated knockdown of Opa1 expression (**Figure 8D**). This finding confirms that when Opa1 expression is sufficiently suppressed, FIR loses its ability to rescue mitochondrial dysfunction via Opa1 activation. This indirectly supports the mechanistic hypothesis that FIR improves mitochondrial function mainly by regulating Opa1.

We also employed the Opa1 inhibitor MYLS22, which blocks mitochondrial fusion (Larrue et al., 2023). **Additional Figure 3D** showed that MYLS22 at concentrations of 1–250 μM had no significant cytotoxic effect on the cells after 24 hours of treatment; therefore, MYLS22 concentration within 250 μM was used for the subsequent experiments. MYLS22 suppressed Opa1 expression (**Additional Figure 3E**). The protective effect of FIR was completely counteracted by the inhibition of Opa1 expression by MYLS22, then it is clear that Opa1 may be a central target of the action of FIR in restoring injury (**Additional Figure 3F**). FIR treatment significantly restored OGD-induced decrease in cell viability, but MYLS22 completely blocked the protective effect of FIR (**Figure 8E**), confirming that Opa1 is a key protein in the neuroprotection exhibited by FIR. This suggests that FIR exerts neuroprotective effects, at least in part, through Opa1 activation, even when mitochondrial fusion is pharmacologically impaired.

## Discussion

In the last 10 years, the efficacy of visible and near-infrared light as therapy modalities for IS has been thoroughly examined (Lapchak, 2010; Choi et al., 2012; Strubakos et al., 2020; Gerace et al., 2021; Yokomizo et al., 2024), particularly in near-infrared studies. Nevertheless, limited research has investigated the therapeutic benefits of FIR on IS. FIR light is progressively used in several biomedical domains because to its negligible negative effects. FIR is a potentially beneficial treatment that needs just low-energy light emitted from a radiator (Tu et al., 2013). FIR irradiation, capable of penetrating up to 1.5 inches beneath the skin, has been documented to yield various beneficial biological effects in both animals and humans, including enhanced blood circulation, mitigation of endothelial dysfunction, stimulation of angiogenesis, reduction of blood pressure, and promotion of capillary dilation (Chen et al., 2022b; Li et al., 2024; Qin et al., 2024). Although FIR appears to exert therapeutic effects in different animal neurological models (Tu et al., 2013; Carlson et al., 2020), there are no clear reports on its beneficial effects on IS. In this study, we firstly used FIR to treat MCAO rats. The results demonstrated that FIR therapy ameliorated brain injury, suppressed cerebral infarction, and improved survival in MCAO rats. Additionally, FIR treatment induced protective effects on the expression of the mitochondrial *Opa1* gene in MCAO rats, suggesting that FIR may have potential to be developed as an alternative therapy for treatment of IS. Although, nimodipine is recognized as an effective treatment for MCAO rats (Carlson et al., 2020), similarly, 30 minutes of FIR improved arthritis symptoms, comparable to the effects observed in the nimodipine-treated group. In MCAO rats treated with FIR, improvements in neurobehavioral scores, the cerebral infarct area, the cerebral edema rate, body weight, survival indices, and rCBF were observed.

A distinguishing feature of our work is the focus on mitochondrial dynamics as a central mechanism. While prior studies have suggested that FIR influences mitochondrial function (Chang et al., 2016; Hsu et al., 2019, 2020; Seo et al., 2021), none have directly linked its benefits in IS to the regulation of mitochondrial fusion and fission. Prior research indicated that the restoration of mitochondrial activity may serve as a method to enhance IS results (Mao et al., 2022; Yang et al., 2022a; Hwang et al., 2024). Ischemic stroke results in a deficiency of oxygen and glucose, which impedes energy metabolism and affects ATP production, thereby causing mitochondrial damage (Jordan et al., 2011; Umbrasas et al., 2021). Decreased ATP levels hinder proper mitochondrial activity, leading to neurological impairment, commonly observed in the brains of IS patients (Jordan et al., 2011). This study revealed that ATP levels in FIR-treated MCAO rats were elevated compared with untreated MCAO rats, likely attributable to enhanced mitochondrial activities following FIR therapy. These findings suggest that FIR treatment partially normalizes mitochondrial function and ameliorates neurological injury, which may be a mechanism for improving neurological outcomes in IS rats. Indeed, disruption of energy homeostasis impairs normal mitochondrial function in IS (Sekerdag et al., 2018; Sprenger and Langer, 2019), and restoring mitochondrial function in the brain has been suggested as a viable approach for treating IS. Moreover, other investigations have demonstrated that FIR diminishes Drp1 expression while enhancing Opa1 and Mfn2 expression in a cellular model of spinocerebellar ataxia (Chang et al., 2016). Of note, we also found that FIR promotes Opa1 expression, thereby affecting mitochondrial kinetic processes. Our works further demonstrated that FIR ameliorated the mitochondrial dysfunction induced *in vitro* and *vivo*, inhibited subsequent mitochondrial division, and promoted mitochondrial fusion as referenced by TEM imaging. Since disruption of mitochondrial kinetic processes in stroke is thought to be due to mitochondrial apoptosis and altered membrane potential generation (Kinnally et al., 2011; Zhang et al., 2020a, b), we therefore hypothesized that FIR might ameliorate mitochondrial apoptosis. Moreover, prior research has demonstrated that Drp1 facilitates the translocation of the pro-apoptotic protein Bax to the mitochondria (Montessuit et al., 2010), altering mitochondrial membrane permeability, changing the mitochondrial membrane potential, releasing cytochrome C, and inducing a cascade reaction leading to apoptosis. Mitochondrial fusion has been shown to save cells from apoptosis (Büeler, 2010). Following our findings, FIR enhanced mitochondrial apoptosis upon OGD induction. For example, FIR therapy partly occluded the mitochondrial permeability transition pore, facilitating the restoration of mitochondrial membrane potential to baseline levels and preserving normal mitochondrial function, thereby averting mitochondrial death.

Nevertheless, there are several limitations to this study. First, representing wavelengths of 4–20 μm as the design of the FIR is a simplification, as these wavelengths cover a broad range (Taylor et al., 2022; Li et al., 2024). Secondly, we examined the impact of FIR on neurological function in MCAO rats at a consistent power density, varying the intervention durations, and concluded that FIR had potential for enhancing neurological function. Nonetheless, the specifications for illumination parameters (e.g., energy density, power density, and treatment duration) may vary for the protective effects of particular light wavelengths (Huang et al., 2009). More systematic studies are needed to determine the suitable light distance and treatment duration for ischemic stroke. Third, FIR light can restore mitochondrial function in cells by altering Opa1 expression. More experiments are required to elucidate the upstream and downstream mechanisms associated with Opa1. Fourth, although the N-2α cell line is a classic model for studying neural energy metabolism and mitochondrial function (Liu et al., 2022), its differences in electrophysiological properties from mature neurons *in vivo* must be interpreted cautiously. In addition to these methodological considerations, certain behavioral findings in our MCAO model also merit careful interpretation. Although the classical MCAO paradigm suggests that rats with left-sided infarction tend to exhibit rightward circling or motor bias, the animals in the present study predominantly showed leftward circling. This discrepancy may be related to the relatively extensive ischemic injury induced by the intraluminal filament MCAO model, which can involve not only the cortex but also motor-related structures such as the striatum and internal capsule (Longa et al., 1989; Belayev et al., 1996; Morris et al., 2016). Given the important role of the striatum in movement direction and rotational behavior, leftward circling in our model may reflect functional imbalance caused by involvement of the left-sided striatum and associated motor circuits (Löscher, 2010; Ruan and Yao, 2020). This behavioural phenotype is further documented in **Additional Videos 1 and 2**.

## Conclusions

We deduce that FIR may confer protective benefits in the MCAO rat model of cerebral ischemia by preventing mitochondrial fission, enhancing mitochondrial fusion, and reinstating normal mitochondrial dynamics and functionality via critical mitochondrial fusion factors such as Opa1. Consequently, as a nonpharmacological and noninvasive intervention, FIR may offer a viable alternative to IS therapy and broaden the range of therapeutic applications to fulfill patients’ expectations.

## Additional files:

***Additional Figure 1:*** *FIR promotes vascular regeneration in Bend3 cells.*

Additional Figure 1 FIR promotes vascular regeneration in Bend3 cells.
(A) Representative images showing the effects of OGD exposure and FIR treatment on tube formation ability of cells. Scale bars: 100 μm. (B) Quantitative analysis and comparison of the tube formation capabilities in all groups. Data are expressed as the mean ± SEM (*n* = 3). ^**^P < 0.01, ^***^P < 0.001. FIR: Far-infrared radiation; Nb: number; OGD: oxygen-glucose deprivation; Tot: total.

***Additional Figure 2:*** *FIR treatment modulates differential gene expression in MCAO rats.*

Additional Figure 2 FIR treatment modulates differential gene expression in MCAO rats.
(A) Differential expression of genes between the MCAO group with or without FIR treatment. Eight genes were upregulated, and 2 genes were downregulated after FIR treatment (*n* ≥ 7). (B) Opa1, Senp3, Aldh2, Parg, Krt75, Omp, Ncf4 gene expression in the cerebral ischemic cortex are verified by quantitative reverse transcription-polymerase chain reaction (*n* ≥ 6). Data are expressed as the mean ± SEM. ^*^P < 0.05, ^**^P < 0.01, ^***^P < 0.001. FIR: Far-infrared radiation; MCAO: middle cerebral artery occlusion; Opa1: optic atrophy 1.

***Additional Figure 3:*** *FIR exhibits protective effect on N-2α cells via activation of Opa1.*

Additional Figure 3 FIR exhibits protective effect on N-2α cells *via* activation of Opa1.
(A) ATP production in each group using siRNA transfection to knock down the Mfn1/2. (B) Knockdown of Opa1 gene expression using siRNA transfection. (C) Knockdown of Opa1 gene expression using siRNA transfection for each treatment group. (D) Cell viability of N-2α cells treated with MYLS22 for 24 hours. (E) N-2α cells were treated with Opa1 inhibitor, MYLS22 (100-200 μM) for 24 hours, and the expression level of Opa1 was measured by western blotting using antibodies against Opa1, β-actin as loading control. (F) N-2α cells were treated with MYLS22 (100 μM) for 24 hours, and the protein expression level of Opa1 was measured by Western blotting using antibodies against Opa1, β-actin as loading control. Data are expressed as the mean ± SEM (*n* = 3). ^*^P < 0.05, ^**^P < 0.01, ^***^P < 0.001. FIR: Far-infrared radiation; Mfn: mitofusin; OGD: oxygen-glucose deprivation; Opa1: optic atrophy 1.

***Additional Table 1:*** *Animal allocation in each experiment.*

***Additional Table 2:*** *Primer sequences used for quantitative reverse transcription-polymerase chain reaction.*

***Additional Table 3:*** *Primer sequences used for mitochondria DNA measurement.*

***Additional Table 4:*** *siRNA sequence.*

***Additional Video 1:*** *Operator A_Left circling.*



***Additional Video 2:*** *Operator B_Left circling.*



***Additional file 1:*** *Proteomic extraction, enzymatic digestion and quantification methods.*

Additional file 1 Proteomic extraction, enzymatic digestion and quantification methods

## Data Availability

*Additional data and materials supporting the findings of this study are available from the corresponding author upon reasonable request.*
