# Supplementary material for: Far-infrared irradiation restores mitochondrial dynamics to ameliorate ischemic stroke
Source: Neural Regen Res. 2026 Jan 27;21(10):4933–44. doi: 10.4103/NRR.NRR-D-25-00399 (PMC13568656; doi:10.4103/NRR.NRR-D-25-00399)
Supplement: Supplementary file 1 [file NRR-21-4933_Suppl1.pdf]

Additional file 1

Proteomic extraction, enzymatic digestion and quantification methods

Total protein extraction

Rat ischemic cortex samples were placed on ice and suspended in protein lysis buffer (8 M urea, 1% sodium dodecyl sulfate [SDS]) containing an appropriate protease inhibitor. The suspension was homogenized using a high-throughput tissue grinder for three cycles of 40 seconds each, followed by incubation on ice for 30 minutes. During incubation, the mixture was vortexed for 5–10 seconds at 5-minute intervals. After centrifugation at 16,000 × g for 30 minutes at 4°C, the supernatant was collected. Protein concentration in the supernatant was determined using the bicinchoninic acid (BCA) assay with a BCA Protein Assay Kit (Thermo Scientific), according to the manufacturer's protocol. Following protein quantification, sodium dodecyl sulfate-polyacrylamide gel electrophoresis was performed.

Protein digestion

Proteins (100 μg) were resuspended in 100 mM triethylammonium bicarbonate (TEAB) buffer. First, the mixture was reduced with 10 mM tris(2-carboxyethyl)phosphine (TCEP) at 37°C for 60 minutes, followed by alkylation with 40 mM iodoacetamide (IAM) at room temperature in the dark for 40 minutes. After centrifugation at 10,000 × g for 20 minutes at 4°C, the pellet was collected and resuspended in 100 μL of 100 mM TEAB buffer. Trypsin was added at a 1:50 (w/w) enzyme-to-protein ratio, and digestion was performed at 37°C overnight.

Peptide desalting and quantification

Following digestion, peptides were dried under vacuum. The dried peptides were then reconstituted in 0.1% trifluoroacetic acid and desalted using an HLB cartridge. The eluted peptides were concentrated again under vacuum. Finally, peptide concentration was determined using a Peptide Quantification Kit (Thermo Fisher Scientific, #23275) according to the manufacturer's instructions.
